# Colostomy

**Published:** 1907-10-26

**Authors:** 


### COLOSTOMY.

The operation of colostomy is usually performed for carcinoma of some part of the large intestine, most often of the rectum, since this viscus is so frequently the seat of malignant disease. But the operation is, at best, a palliative measure, and it must not be thought that its performance is indicated in all cases of carcinoma of the rectum. Radical treatment by excision of the disease is the proper procedure whenever it is feasible: the surgeon must first make up his mind on this point. The factors which he must take into consideration are: (1) Is the growth fixed to surrounding structures? If on digital examination of the rectum the mass felt can be moved in all directions, it is fair to assume that it has not yet involved by direct extension the base of the bladder or the structures lying between it and the sacrum. If this has already occurred successful excision is impossible. (2) Are there any secondary growths in other abdominal viscera? The liver is the organ which is principally affected. This is easy to understand, because the superior hæmorrhoidal vein is the radicle of the inferior mesenteric, and thus pours its contents into the portal circulation. There is no doubt that cancer cells are thus conveyed in this way to the liver, and, owing to their peculiar power of reproducing in new situations the structure from which they are derived they give rise to metastatic deposits. In view of the indisputable fact that involvement of the liver occurs by means of the portal vein, the old idea that carcinoma could only spread by the lymphatic system must be finally abandoned. In early cases it is impossible to detect involvement of the liver by palpation of the abdominal wall; but when the disease is advanced the organ is enlarged and studded on its surface with palpable protuberances whose centre is depressed—a condition to which the term " umbilication " is applied. Some authorities argue that metastasis in the liver is not necessarily a contraindication to excision of the growth, because the secondary growths increase less rapidly than the original focus. In the writer's opinion it is more reasonable to assume that if secondary deposits in the liver have attained such dimensions as to be palpable through the abdominal wall the prospect of benefiting the patient by radical treatment is extremely remote, and to regard such a condition as a definite contraindication to excision of the rectum. (3) Is the patient's general condition satisfactory? Excision of the rectum is an operation which is associated with much shock, which may cause a fatal result in a patient already debilitated by malignant cachexia.

In cases in which a radical operation is contraindicated careful attention must be paid to the patient's diet. He should subsist, as far as possible, on foods which have no residue, so that it is not necessary to open the bowels more than once in three or four days. This is a point of great importance to his comfort, since the act of defæcation causes great pain. He should, of course, be kept under observation, so that colostomy may be performed as soon as there is any definite indication for it.

What are the indications? The most important is obstruction. But even this does not always demand colostomy. When the symptoms of obstruction make their appearance a digital examination should be made; and if the rectum is found to be obstructed by a large friable growth sprouting into its lumen, it is better treatment to scrape away the mass than to do a colotomy, provided the sphincter ani is still functional. For it must always be remembered that a patient is happier without a colostomy than with one. But if the obstruction is due to a tight annular stricture there is no choice open to the surgeon but to perform a colostomy. Another definite indication is the constant passage of an acrid discharge from the growth combined with loss of sphincteric power, which renders the patient's life intolerable to him. Colostomy will relieve this by preventing the passage of fæcal matter over the diseased area, and will greatly diminish, if it does not altogether arrest, the discharge. It has been said that severe pain in carcinoma of the rectum should be regarded as an indication for colostomy, but the statement should be received with caution. The pain is generally due to direct involvement of one or other of the nerve trunks in the pelvis by the disease rather than to the passage of irritating material down the rectum; and colostomy is less likely to relieve this than is morphia.

The indications for the performance of colostomy in carcinoma of the rectum have been dealt with at some length, because it is for this disease that the operation is most often performed. But it is also called for in cases of intestinal obstruction due to inoperable carcinoma in any part of the large intestine; although the site of the colostomy must vary in any given case according to the situation of the stricture —that is, it must be performed in such a situation that the gut is opened on the proximal side of the stricture. If this precaution is not observed, operative interference is worse than useless.

Other conditions which may demand this operation are (1) syphilitic or tuberculous ulceration of the lower end of the rectum, which has resisted palliative treatment by dilatation with bougies or by linear proctotomy, (2) the more advanced degrees of congenitally imperforate anus (the outlook in these cases is not promising), or (3) recto-vaginal fistula resulting from damage to the parts during parturition. Finally, it may be done as a preparatory measure before excising the rectum.

*(To be continued.)*

